# Broad Spectrum Anti-Bacterial Activity and Non-Selective Toxicity of Gum Arabic Silver Nanoparticles

**DOI:** 10.3390/ijms23031799

**Published:** 2022-02-04

**Authors:** Adewale O. Fadaka, Samantha Meyer, Omnia Ahmed, Greta Geerts, Madimabe A. Madiehe, Mervin Meyer, Nicole R. S. Sibuyi

**Affiliations:** 1 Department of Science and Innovation (DSI)/Mintek Nanotechnology Innovation Centre (NIC), Biolabels Research Node, Department of Biotechnology, University of the Western Cape (UWC), Bellville 7535, South Africa; afadaka@uwc.ac.za (A.O.F.); amadiehe@uwc.ac.za (M.A.M.); 2 Department of Biomedical Sciences, Faculty of Health and Wellness Sciences, Cape Peninsula University of Technology, Bellville 7535, South Africa; meyers@cput.ac.za; 3 Department of Restorative Dentistry; University of the Western Cape, Bellville 7535, South Africa; 3689306@myuwc.ac.za (O.A.); ggeerts@uwc.ac.za (G.G.)

**Keywords:** anti-bacteria, cytotoxicity, green synthesis, gum arabic, silver nanoparticles

## Abstract

Silver nanoparticles (AgNPs) are the most commercialized nanomaterials and presumed to be biocompatible based on the biological effects of the bulk material. However, their physico-chemical properties differ significantly to the bulk materials and are associated with unique biological properties. The study investigated the antimicrobial and cytotoxicity effects of AgNPs synthesized using gum arabic (GA), sodium borohydride (NaBH_4_), and their combination as reducing agents. The AgNPs were characterized using ultraviolet-visible spectrophotometry (UV-Vis), dynamic light scattering (DLS), transmission electron microscopy (TEM), and Fourier-transform infrared spectroscopy (FT-IR). The anti-bacterial activity was assessed using agar well diffusion and microdilution assays, and the cytotoxicity effects on Caco-2, HT-29 and KMST-6 cells using MTT assay. The GA-synthesized AgNPs (GA-AgNPs) demonstrated higher bactericidal activity against all bacteria, and non-selective cytotoxicity towards normal and cancer cells. AgNPs reduced by NaBH_4_ (C-AgNPs) and the combination of GA and NaBH_4_ (GAC-AgNPs) had insignificant anti-bacterial activity and cytotoxicity at ≥50 µg/mL. The study showed that despite the notion that AgNPs are safe and biocompatible, their toxicity cannot be overruled and that their toxicity can be channeled by using biocompatible polymers, thereby providing a therapeutic window at concentrations that are least harmful to mammalian cells but toxic to bacteria.

## 1. Introduction

In the past, silver-based compounds were used as antimicrobial agents due to their microbicidal activities [1]. Their biomedical application was encouraged by the fact that silver ions (Ag^+^) and their related compounds are less toxic towards mammalian cells while being highly toxic to microorganisms, such as bacteria and fungi [2,3]. Recent advances in the field of nanotechnology have influenced and increased the use of silver-based compounds at a nanometer size. Several physical and chemical methods have been reported for the synthesis of AgNPs [4,5], however, AgNPs produced by these methods lead to the production of noxious compounds that are toxic to cells and the environment. To overcome these toxic effects, green synthesis methods, using natural products as reducing and stabilizing agents, were developed [6]. Green synthesis methods produce nanoparticles (NPs) using eco-friendly and non-toxic biological agents, such as microorganisms (e.g., bacteria, yeasts, fungi, and algae) and plant extracts as reducing and stabilizing agents [1,7,8]. Plant-extract mediated green synthesis of NPs is often preferred over the microbial-mediated synthesis method due to the biohazards and laborious process associated with the latter [9,10]. The use of plant extracts in green synthesis is easier, more efficient, eco-friendly and incurs low cost in comparison with the chemical or microbial mediated synthesis methods. Plant materials are cost-effective as plants are renewable, readily available, and contain antioxidant-rich phytochemicals [4] that can play a major role in the reduction and stabilization of Ag^+^ into bioactive AgNPs. The availability of plants makes the green method amenable to large-scale production of NPs. Over the last few years, there has been an upsurge in the application of plant-extract-reduced AgNPs on account of their immense antimicrobial efficacy, and they are perceived as future-generation therapeutic agents against drug-resistant microbes. Examples of plant-based AgNPs that have demonstrated good anti-bacterial properties and potential anticancer effects include those synthesized using *Chrysanthemum indicum L* [11], *Acacia leucophloea* [12] and *Ganoderma neojaponicum Imazeki* [13] extracts.

AgNPs have distinct and superior properties compared to their bulk materials, and this has afforded their integration into numerous consumer (e.g., cosmetic and household) and health products to prevent microbial infestation and growth. AgNPs are now present in commercial products used daily, such as toothpaste, sunburn lotions, food packaging, medical devices, and clothing [14,15]. In addition to the antimicrobial effects of AgNPs against infectious microbes [16], they are used in catalysis [17], disease treatment [18,19], and as additives in polymerizable dental material [20,21,22].

Polysaccharides have played a huge role in the application of nanomaterials, especially in biomedical applications. Polysaccharides derived from algae (*Pterocladia capillacae*, *Jania rubins*, *Ulva faciata*, and *Colpmenia sinusa*) [23] and plants (gum arabic, GA [24]) alike, were previously used as stabilizers and capping agents for nanomaterials, both chemical and green synthesized NPs, to enhance their biocompatibility and biosafety. The most widely explored polysaccharide-rich compounds are chitosan [25] and GA [24]. GA is a natural plant-based gum composed of a complex mixture of glycoproteins and polysaccharides, in addition to being a historical source of monosaccharides, arabinose and ribose. GA is considered a safe additive with no adverse effects [26] and has wide applications in the food (e.g., stabilizer, thickening agent and hydrocolloid emulsifier), textile (e.g., pottery, lithography, and cosmetics) and pharmaceutical industries [27]. In the field of nanotechnology, GA has been employed because of its biocompatibility and stabilization effects for nanomaterials [28,29], such as iron oxide NPs [30,31,32], gold nanoparticles (AuNPs) [33,34,35], carbon nanotubes [36], quantum dots [37], AgNPs [24], and chitosan NPs (CT-NPs). Cross-linking the carboxylic groups of GA with CT produced CTGA-NPs that had improved mechanical properties, and which consequently found application as a bone graft substitute for bone regeneration [38]. GA has also been used as a reducing agent for the synthesis of GA-AgNPs [6,39]. GA-AgNPs showed potential as promising candidates in the development of antioxidant, anti-inflammatory, antimicrobial [6] and anticorrosive agents [40]. This study demonstrated the anti-bacterial and cytotoxicity effects of AgNPs green-synthesized using GA.

## 2. Results and Discussion

GA is a non-toxic glycoprotein polymer commonly used as a stabilizer in the food and pharmaceutical industries. It has various pharmacological properties; apart from being used as an emulsifying agent, it has antioxidant, anti-diabetic, and anti-lipid peroxidation properties, among others [41,42]. The chemical composition of GA is complex and varies among species, where all have high levels of carbohydrates and very low protein content [43].

The GA species used in the current study (*Acacia senegal*) had negligible flavonols, flavanols, TPC, with no antioxidant, radical scavenging or reducing abilities at 4 mg/mL, as shown in Table 1. As such, GAE on its own was incapable of reducing a metal precursor into metallic NPs at temperatures ≤100 °C. Due to its high sugar content, solubility and binding capacity, GA has been used as a stabilizer for AuNPs [24,30]. It stabilizes NPs by binding to other biomolecules on their surface through its abundant carboxyl groups [30].

### 2.1. Synthesis of GA-AgNPs

Synthesis of GA-AgNPs was first attempted at R_T_ and by boiling (~100 °C) solutions that contained 4 mg/mL GAE and various AgNO_3_ concentrations (1–5 mM). No GA-AgNPs were formed at all the tested concentrations; there was no color change in the solution at R_T_ and a pinkish color was observed after boiling the solution (data not shown). The negligible phytochemical and lack of antioxidant contents reported for GAE in Table 1 provides a clear indication that the GAE at the concentration used in the current study was incapable of reducing AgNO_3_ to form GA-AgNPs. Other studies attempted to synthesize GA-AgNPs by devising methods to potentiate the reducing abilities of GAE, by changing the GAE pH [44], and by using honey as a reducing agent, while using GA as a stabilizer for the AgNPs [40].

A novel, greener approach, using an autoclave method, was established for other gum species to produce sterile AgNPs without additional reducing agents or change of pH. This method was successful in the reduction of AgNO_3_ by gum acacia [39], gum tragacant [45] and *piyar* gum [46]. The same method was adapted for the synthesis of GA-AgNPs in the current study and was optimized by first varying the concentrations of AgNO_3_ (0.1–0.5 g/40 mL) then the GAE concentrations (2–6 mg/mL/40 mL). The optimized conditions (i.e., concentrations of GAE and AgNO_3_) were further used in the combined approach, with NaBH_4_ as an additional reducing agent, to synthesize GAC-AgNPs.

**Figure 1:**
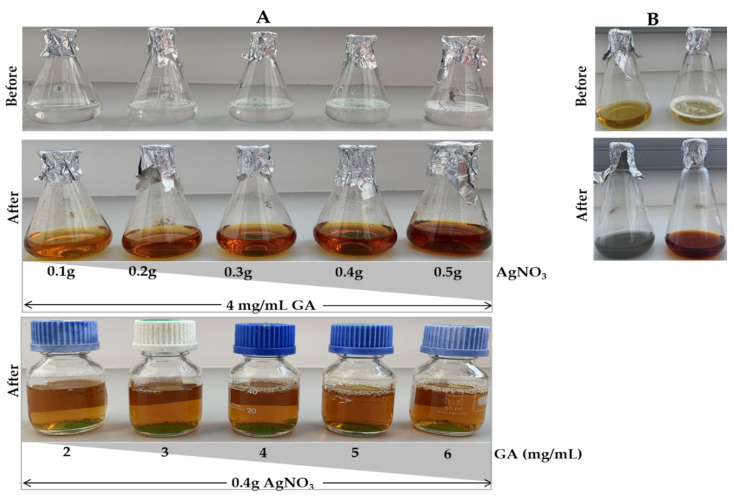
Synthesis of GA-AgNPs using green (**A**), chemical and combined (**B**) approaches.

Using the green synthesis approach, the solution containing GAE and AgNO_3_ was colorless before autoclaving and changed to brown after autoclaving (Figure 1A). The color intensity increased with increasing concentrations of AgNO_3_ and GAE. In the combined approach, the samples turned yellow immediately after adding ice cold AgNO_3_, then to a grayish green color for the C-AgNPs, and brown for the GAC-AgNPs (Figure 1B) after autoclaving. Based on the colors, the GAC-AgNPs were more stable than the C-AgNPs.

The color change was a first indication of formation of the AgNPs, which are reported to have yellow, orange or brown colors [46,47]. Thus, the brown color indicated that GAE at high temperature (120 °C) and pressure (15 psi) was able to reduce Ag^+^ into Ag^0^, and form GA-AgNPs and GAC-AgNPs. The GAE in the green synthesis approach acted as both a reducing and capping agent for the GA-AgNPs. It is very common in green synthesis, especially for plant-derived NPs, for the biomolecules found in the extracts to serve as reducing, capping and stabilizing agents [47,48]. Plants contain a lot of phytochemicals (e.g., alkaloids, flavonoids, terpenoids, etc.), enzymes/proteins, amino acids, polysaccharides, and vitamins, that can aid in the reduction of metal salts in a rapid and environmentally benign process. Green synthesis is quite advantageous, as it is cost-effective and can be easily scaled up to produce biocompatible AgNPs. Moreover, the medicinal efficacy of the extracts will be a valuable addition to the NPs and enhance their pharmacological activities [40,47,49].

### 2.2. Characterization of the AgNPs

#### 2.2.1. Optical Properties of the AgNPs

**Figure 2:**
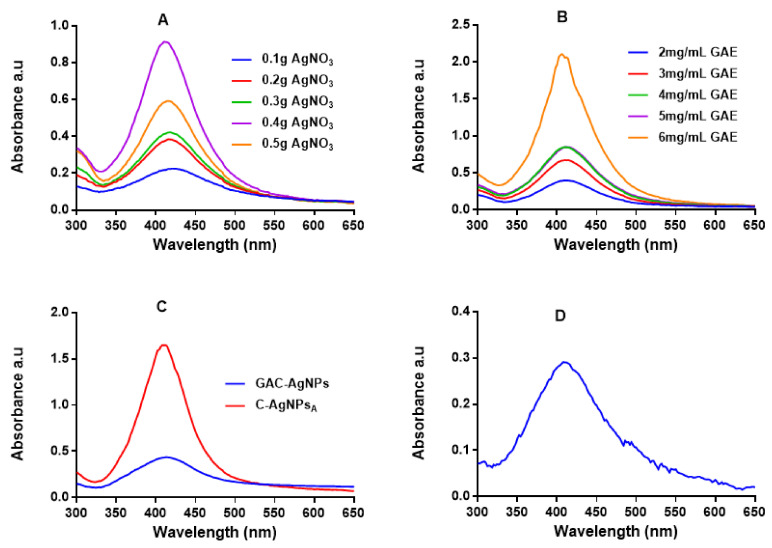
UV-Vis analysis of the AgNPs. The synthesis of GA-AgNPs was optimized using varying concentrations of AgNO_3_ and 4 mg/mL GAE (**A**), varying GAE concentration and 0.4 g AgNO_3_ (**B**), and by using NaBH_4_ alone and in combination with 4 mg/mL GAE (**C**); all these solutions were autoclaved at 121 °C. C-AgNPs were synthesized at 70 °C (**D**).

UV-Vis spectrophotometry was used to confirm the formation of the AgNPs, which have a characteristic SPR around 400 nm [47,49]. Figure 2 shows the absorption spectra for the AgNPs produced via the green (GA-AgNPs), chemical (C-AgNPs) and combined (GAC-AgNPs) approaches. All the concentrations of GAE and AgNO_3_ were able to synthesize AgNPs, which was confirmed by a characteristic SPR for AgNPs at ~400 nm. The peak intensity of the GA-AgNPs synthesized with 0.4 g AgNO_3_ was higher than all the other concentrations (Figure 2A), which suggested that more AgNPs were formed at this concentration [48]. An amount of 0.4 g AgNO_3_ was selected as an optimum concentration and used to optimize the concentration of GAE (2–6 mg/mL). The optimum GAE concentration was 4 mg/mL; both 4 and 5 mg/mL of GAE gave a similar spectral profile, indicating that GA-AgNPs of the same yield, size and shape were produced by the two concentrations (Figure 2B). Although 6 mg/mL showed higher biomass compared to all the GAE concentrations, there were some black precipitates after autoclaving the sample. The precipitates might have contained excess GAE and indicated that the extract concentration might be too high.

In the combined approach, AgNO_3_ was reduced in the presence of GAE and a chemical reducing agent (NaBH_4_) to produce GAC-AgNPs (Figure 2C). There are two assumptions as to how the GAC-AgNPs were produced, the first involves NaBH_4_ acting as a reducing agent to form C-AgNPs (before autoclaving) which are then capped/stabilized by GAE to form GAC-AgNPs during the autoclave process. The second assumption is that GAE and NaBH_4_ might have acted synergistically as reducing agents. The differences in the spectral profiles of the C-AgNPs_A_ and GAC-AgNPs (Figure 2C) might have occurred as a result of the instability of C-AgNPs when exposed to high temperatures. The C-AgNPs synthesized at 70 °C were used in further studies (Figure 2D).

#### 2.2.2. Morphology and Size Distribution of the AgNPs

**Figure 3:**
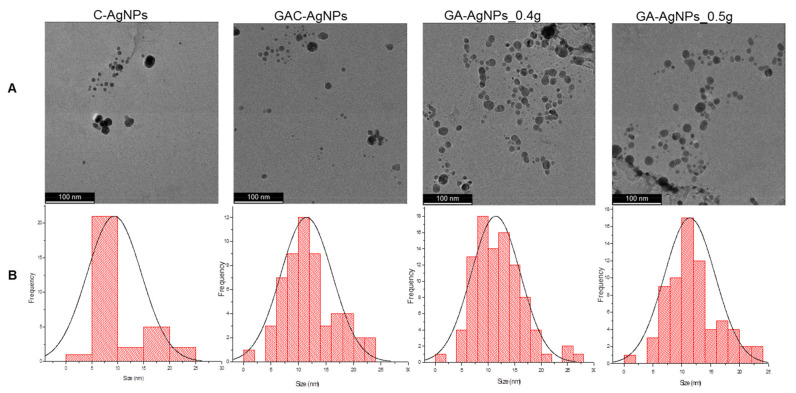
HRTEM micrographs of the AgNPs (**A**) and the AgNP core size distribution (**B**).

The morphology and core size of the AgNPs were analyzed by HRTEM. As shown in Figure 3A, the majority of the AgNPs were spherical in shape; their core size distribution varied from 1–30 nm.

DLS analysis revealed a hydrodynamic diameter range from 87.22 nm for the C-AgNPs to 94.62 nm for the GA-AgNPs to 144.39 nm for the GAC-AgNPs (Table 2). These sizes vary from those obtained from the HRTEM as they account for both the core size and the molecules on the surface of the AgNPs [47]. The C-AgNPs had a smaller hydrodynamic size, followed by the GA-AgNPs, while the GAC-AgNPs were the largest in size. This indicates that the GAE played a crucial role in the synthesis of the GA-AgNPs as both reducing and capping agents.

All the AgNPs had a negative zeta (ζ) potential, except for the GAC-AgNPs (9.33 mV). The polydispersity index (Pdi) indicated that GA-AgNPs, followed by C-AgNPs, were the most stable. Pdi serves as an indicator for the dispersity and stability of NPs, thus, NPs with a Pdi that is ≤0.05 are regarded as stable and monodispersed. Materials with a Pdi of ≥0.7 are classified as polydispersed, with broad size distribution and being less stable in suspension [47]. The GA-AgNPs_0.4g and GA-AgNPs_0.5g demonstrated similar physicochemical properties (Table 2), and the two were investigated further to determine if they have similar bioactivities as well.

#### 2.2.3. FT-IR Analysis of GAE and AgNPs

FT-IR was used to identify the functional groups in GAE and those that were involved in the intermolecular interactions between the precursor (AgNO_3_) and reducing agents (NaBH_4_ and GAE). The intermolecular interactions between the samples occurs via hydrogen bonding or dipole–dipole interactions during synthesis and cause shifts in the frequency or absorption of the functional groups [50] that can be assigned to a particular biomolecule.

**Figure 4:**
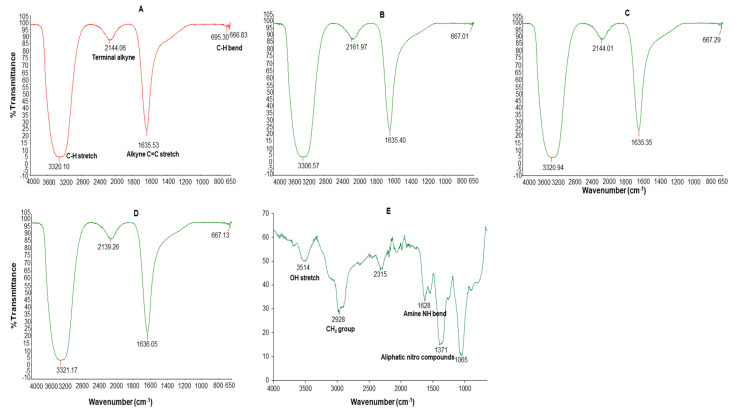
FT-IR spectra of C-AgNPs (**A**), GA-AgNPs_0.4g (**B**), GA-AgNPs_0.5g (**C**), GAC-AgNPs (**D**), and GAE (**E**).

The dominant absorption bands at 3306–3321, 2139–2161, 1635–1636 and 695–667 cm^−1^ were identified in the FT-IR spectrum of all the AgNPs (Figure 4). These bands were associated with the alkyne C-H stretch (3320–3310), terminal alkyne monosubstituted (2140–2100), C≡C stretch (2260–2100), alkenyl C=C stretch (1680–1620), amide (1680–1630), secondary amine NH bend (1650–1550), alkyne C-H bend (680–610), organic nitrates (1640–1620), and aromatic C-H out-of-plane bend (900–670) [51].

The GAE FT-IR spectra had five major absorption peaks at 3514 cm^−1^ (3570–3200 cm^−1^ OH stretch), 2978 (CH_2_ group in aliphatic chains), 2315, 1628 cm^−1^ (1650–1550 cm^−1^ secondary amine NH bend), and 1371 cm^−1^ (1380–1350 aliphatic nitro compounds) and 1065 cm^−1^. The presence of different functional groups was a reflection of the phytochemical composition of the GAE; the OH bonds are attributed to alcohols or phenols and the N–H bond to amides which might be from the carbohydrates and proteins in GAE. The GAE FT-IR peaks showed similarity to those of other GA species, such as *Acacia senegal* and *Acacia seyal* [40,50,52]. Thus, the carbohydrates and proteins in GAE were responsible for the reduction, capping and stabilization of the GA-AgNPs and possibly the GAC-AgNPs.

### 2.3. Stability of GA-AgNPs

**Figure 5:**
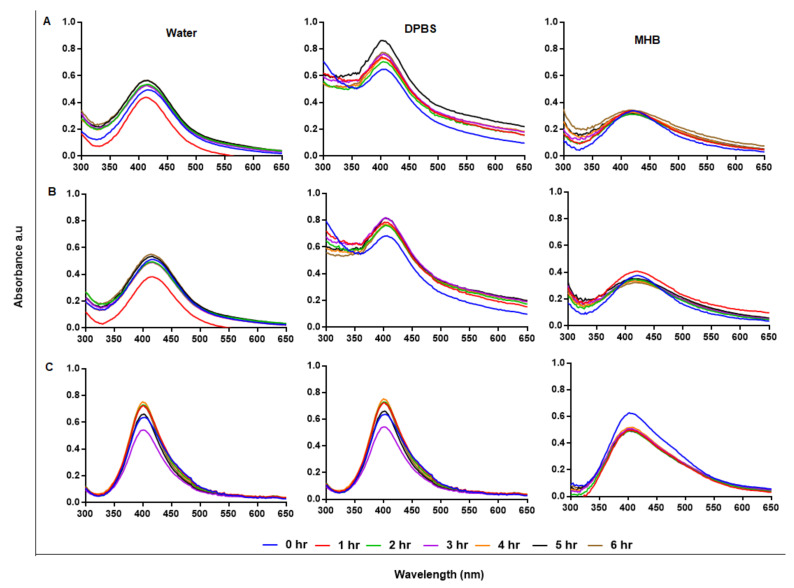
Assessment of AgNP stability in solution by UV-Vis. The GA-AgNPs_0.4g (**A**), GA-AgNPs_0.5g (**B**) and GAC-AgNPs (**C**) were diluted in water, DPBS and MHB and incubated at 37 °C for 1–6 h.

Stability of NPs in solutions other than water is crucial for bio-applications and requires NPs that can retain their physical characteristics when introduced into a biological environment. AgNPs are usually very stable in water; however, water is hypotonic and not a suitable vehicle for bioassays [53]. In addition to Pdi, the stability of AgNPs in suspension can also be predicted by their UV-Vis spectral profiles with a characteristic SPR at 400 nm [48]. AgNPs that are not stable will be recognized by aggregation or precipitation out of solution, and if the AgNPs precipitate they will not be useful as antimicrobial agents, as Ag^+^ are known for this effect and have been used for the same purposes [54]. Stability of the AgNPs was assessed at hourly intervals for 6 hr after incubation at 37 °C, as shown in Figure 5A–C; the AgNPs were relatively stable in water, DPBS, and Mueller–Hinton broth (MHB). Cellular uptake of AgNPs is time and size dependent, where uptake and internalization of AgNPs by mammalian cells can occur within 0.5 h [55]. Following the growth kinetics of *Burkholderia pseudomallei,* the interaction and uptake of AgNPs by the bacterial species could be rapid, as the bacteria were killed within 5 min [56]. Biological assays, such as bacteria and cell culture, are performed at 37 °C and AgNPs can be used in culture media for bioassays without aggregation [57]. The components in the media can interact with the AgNPs and change their physicochemical properties and activity; hence, the AgNP-media interactions must be assessed to confirm NP stability before evaluating their activity [58]. Subjecting AgNPs to solutions with higher salt (NaCl) concentration, not only causes NP aggregation, but also a change in size and biological activity [59]. To improve on AgNP stability, biopolymers, such as GA and chitosan, were used as stabilizing agents; this led to plant-mediated synthesis of NPs with enhanced stability, biocompatibility and biological activity.

### 2.4. Anti-Bacterial Activity

Microbial resistance is among the leading factors responsible for death worldwide, due to the overwhelming abuse and misprescription of antibiotics [60,61]. Over the years, alternative antimicrobial agents effective against resistant strains have continually been sought [62,63]. Among others, AgNPs have displayed broad spectrum antimicrobial effects, even against multi-drug-resistant microbes. Of interest are the AgNPs produced through green synthesis, which are presumed to be biocompatible since they are reduced and coated by natural products [47,48,49,62,64].

The anti-bacterial effects of GAE and AgNPs were evaluated on Gram-positive (*S. aureus*, *MRSA*, *S. epidermidis*, *S. pyogenes*) and Gram-negative (*K. pneumoniae, E. coli*) bacteria. The susceptibility of the bacteria to the treatments was assessed through agar well diffusion and broth microdilution methods. Agar well diffusion demonstrated a lack of clearing zones (zone of inhibitions, ZOIs) in the bacteria that were exposed to MHB (negative control), GAE and GAC-AgNPs, indicating lack of anti-bacterial activity (Table 3) at the concentrations used in this test. Anti-bacterial activity of GAE was reported at concentrations ≥40 mg/mL for various GA species [65], while organic solvent GA extracts were effective from 0.25 to 2 mg/mL [66]. The GA-AgNPs and the C-AgNPs showed potency against the selected bacteria, both Gram-positive and Gram-negative strains; the highest anti-bacterial activity was observed with the GA-AgNPs when compared to the C-AgNPs. The two GA-AgNPs exhibited similar activity against the test bacteria. Similar effects were reported for GA-AgNPs synthesized using other GA species; the GA-AgNPs were potent against oral (*Streptococcus mutans*) [67] and fish (*Aeromonas hydrophila* and *P*. *aeruginosa*) [68] pathogens. The activity of the GA-AgNPs in these pathogens was size, as well as concentration, dependent. C-AgNPs capped with citrate were reported to show size-dependent activity against *E*. *coli* and *S*. *aureus* [69].

The MICs of the treatments were visually evaluated on the bacteria following microdilution assay. After 24 h treatment, GAE, C-AgNPs and GAC-AgNPs were unable to inhibit growth at all tested concentrations (6.25–100 µg/mL), as shown in Table 4. Bacterial growth inhibition was observed at 6.25–100 µg/mL for the two GA-AgNPs for all strains, with an MIC of 6.25 µg/mL, except for GA-AgNPs_0.4g effect in *E. coli* which had an MIC of 25 µg/mL. The MIC values were consistent with the GA-AgNPs reported by other studies; the NPs had an MIC of 10 µg/mL in *S*. *mutans* [67], 11–45 µg/mL in *P*. *aeruginosa* [44], 1.625 and 3.25 µg/mL for *A*. *hydrophila* and *P*. *aeruginosa*, respectively [68]. The results in the current study were further confirmed by the Alamar Blue assay, which quantifies the metabolic activity of cells. Only live bacteria can convert the blue resazurin dye into a pink and fluorescent resorufin. The color/fluorescent intensity is directly proportional to live bacteria [62,70].

**Figure 6:**
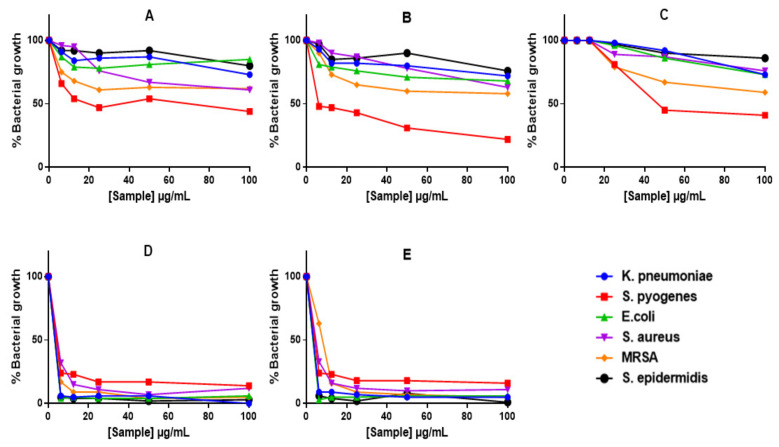
The anti-bacterial effects of GAE and AgNPs using Alamar Blue assay. Bacteria were treated with GAE (**A**), GAC-AgNPs (**B**), C-AgNPs (**C**), GA-AgNPs_0.4g (**D**), and GA-AgNPs_0.5g (**E**).

Alamar Blue assay demonstrated reduction in bacterial growth with all treatments (Figure 6), including those that did not show ZOIs or MICs (i.e., GAE, C-AgNPs and GAC-AgNPs). The GAE showed stronger activity against Gram-positive bacteria, *S. pyogenes, MRSA* and *S. aureus* (Figure 6A). In contrast, *S. epidermidis* and the Gram-negative bacteria displayed some resistance towards these treatments. The GA-AgNPs were consistent in their activity, with significant effects being observed against all the strains above 6.25 µg/mL (Figure 6D,E).

The effects of GAE, C-AgNPs and GAC-AgNPs were not bactericidal, and their MBC values were undetermined. The GA-AgNPs had bactericidal effects on >60% of the selected strains, with MBCs ranging between 12.5 and 100 µg/mL (Table 5). The GA-AgNPs were active against the Gram-positive and Gram-negative bacteria and demonstrated similar trends in both antibiotic susceptible and resistant strains. This is a desirable property and implies that these NPs can be used as broad-spectrum anti-bacterial agents.

### 2.5. In Vitro Cytotoxicity of GA-AgNPs

AgNPs have demonstrated unique properties compared to their bulk counterparts, and these have raised many concerns for biomedical application due to their ability to cross all cellular barriers and interact with important cellular organelles, such as the mitochondria and nucleus [7]. When inside cells, AgNPs can react with biomolecules, such as nucleic acids, proteins, enzymes, etc., resulting in dissolution and release of Ag^+^. The Ag^+^ are presumed to be responsible for the toxicity of the AgNPs [3].

**Figure 7:**
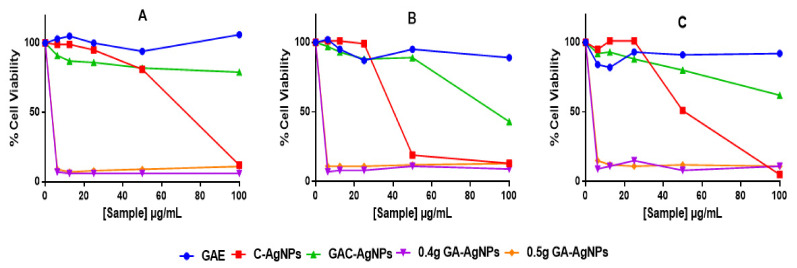
Cytotoxicity of the GAE, C-AgNPs, GAC-AgNPs and GA-AgNPs on KMST-6 (**A**), Caco-2 (**B**) and HT-29 (**C**) cells.

The cytotoxicity of the AgNPs was investigated *in vitro* in two colon cancer (Caco-2 and HT-29) and non-cancerous (KMST-6) cells using 3-(4,5-dimethylthiazol-2-yl)-2,5-diphenyltetrazolium bromide (MTT) assay. The assay quantifies live cells by evaluating their mitochondrial metabolic activity, where live cells are able to reduce the MTT salt into the water-insoluble purple formazan. The color intensity of the dimethyl sulfoxide (DMSO)-dissolved formazan, which is measured by a spectrophotometer, is directly proportional to the amount of live cells [71]. As shown in Figure 7, GAE exhibited insignificant effects on the three cell lines. Of the four AgNPs, GAC-AgNPs were least toxic and showed selective effects to the non-cancer cells. Significant effects of GAC-AgNPs were observed on cancer cells at ≥50 µg/mL. The C-AgNPs were toxic to all cells at ≥50 µg/mL. GA-AgNPs were non-selective and were toxic against both cancer and non-cancer cells, with <15% viable cells at all concentrations.

Therapeutic agents are deemed biocompatible when they have selective toxicity towards diseased cells or are at least cytotoxic at concentrations that are not toxic to normal cells. However, this was not the case with the GA-AgNPs, as these NPs were extremely toxic and nonspecific. The GA-AgNPs had an IC_50_ (Table 6) that was >5-fold lower than their MIC and MBC. Their toxicity was even higher to the non-cancer cells than the cancer cells, with the IC_50_ values of 0.67 µg/mL on the KMST-6 cells and 0.82–1.16 µg/mL on the colon cancer cells.

The anti-bacterial effects of AgNPs have led to their use in several consumer and medical products. With an increased exposure rate to consumers who use, handle or manufacture these products, AgNPs can easily accumulate in human organs via inhalation, transdermal absorption, and ingestion [72]. Although it is known that over exposure to silver salts cases argyria [73], the chronic effects of AgNPs are still elusive and still under investigation. Based on their physicochemical properties, the biological effects of AgNPs can vary. Many studies have reported the biocompatibility, as well as toxicity, of AgNPs in *in vitro* and *in vivo* models [15,16,74]. The toxicity of AgNPs, which is often attributed to the leaching of Ag^+^ [75], has been demonstrated to be size and cell-specific [76]. Green synthesized *Annona muricata*-AgNPs were only toxic to acute monocytic leukemia (THP-1) and breast cancer (AMJ-13) cells, while sparing the normal breast epithelial (HBL) cells [74]. Poly(*N*-vinyl pyrrolidone)-coated AgNPs were not toxic to T cells at concentrations up to 50 ppm, but induced cell death of human mesenchymal stem cells (hMSCs) and monocytes at 30 and 50 ppm, respectively [3]. The anti-cancer properties of AgNPs were shown in several cancer cell lines; however, their toxicity towards both healthy and diseased cells is a huge concern for human health, as these NPs accumulate in biologically important organs, such as the liver, spleen, lung, kidney, and brain. Moreover, smaller size AgNPs are more toxic than larger sizes and surface coatings can be used to defer or control their activity [77].

## 3. Materials and Methods

### 3.1. Preparation of the GA Extracts

The GA extract (GAE) was prepared by dissolving a required amount of GA obtained from *Acacia senegal* (North Kordofan, Sudan) in hot water and filtering through 0.45 µm filters. The GAE was prepared fresh before use.

#### Phytochemical Analysis and Antioxidant Capacity

The amount of flavanols, flavonols, total polyphenolic content (TPC) and antioxidant capacity, was assessed using the ferric reducing antioxidant power (FRAP) assay kit (Sigma, St. Louis, MO, USA). An oxygen radical absorbance capacity (ORAC, Sigma) assay and 2,2-diphenyl-1-picrylhydrazyl (DPPH, Sigma) assay of 4 mg/mL GAE was quantified following standard biochemical methods as previously described [78].

### 3.2. Synthesis of AgNPs

The AgNPs were synthesized through: (a) chemical synthesis (C-AgNPs), (b) green synthesis (GA-AgNPs), and (c) a combined approach (GAC-AgNPs). All solutions were prepared in double-distilled water.

#### 3.2.1. Chemical Synthesis

C-AgNPs were prepared following a previously described method with few modification [79]. Briefly, 10 mL of 2 mM NaBH_4_ (Sigma) was added to 30 mL of double-distilled water and heated to 70 °C on a heating mantle while stirring. The solution was stirred vigorously at 250 rpm. Subsequently, 20 mL of 1 mM ice-cold silver nitrate (AgNO_3_, Sigma) was added dropwise into NaBH_4_ solution. The solution was removed from the heating mantle after a color change to yellow/brown and cooled to room temperature (R_T_, ~25 °C).

#### 3.2.2. Green Synthesis

GA-AgNP synthesis was adapted from a method by Venkatesham et al. [39]. A fixed concentration of GAE (4 mg/mL) was used to prepare GA-AgNPs by varying concentrations (0.1–0.5 g) of AgNO_3_ in a final volume of 40 mL double-distilled water. The method was repeated, keeping the AgNO_3_ (0.4 g) constant and varying the concentration of GAE (2–6 mg/mL). The samples were autoclaved at 121 °C and 15 psi for 20 min and removed after 60 min when the pressure had reduced to 0 psi.

#### 3.2.3. Combined Approach

GAC-AgNPs were synthesized following the green synthesis method (Section 3.2.2) in a reaction mixture comprised of 20 mL of 1 mM silver AgNO_3_, 4 mg/mL GAE and 10 mL of 2 mM NaBH_4_ in a final volume of 40 mL. The synthesis was carried in the autoclave, as described in Section 3.2.2.

All the AgNPs (C-AgNPs, GA-AgNPs, and GAC-AgNPs) were washed twice and harvested by centrifugation at 9000 rpm for 30 min. The pellets were resuspended in double-distilled water and stored in amber bottles at R_T_ in the dark.

### 3.3. Characterization of the AgNPs

#### 3.3.1. UV-Visible Spectrophotometer

The formation of AgNPs was monitored by measuring the UV-Vis spectrum of the reaction medium in the wavelength range from 300 to 650 nm using a POLARstar Omega plate reader (BMG LABTECH, Offenburg, Germany).

#### 3.3.2. Dynamic Light Scattering (DLS)

The hydrodynamic size, surface charge, and Pdi of the AgNPs were analyzed by a Malvern NanoZS90 Zetasizer (Malvern Panalytical Ltd., Enigma Business Park, UK). The synthesized AgNPs were diluted 5-fold with double-distilled water; 1 mL aliquots were sampled in DLS cuvettes or DS1070 zeta cells (Malvern Panalytical Ltd.) and examined for size distribution and zeta potential, respectively. The particle diameters were assessed at a scattering angle of 90 °C at R_T_. The data were represented as mean particle diameter of three measurements.

#### 3.3.3. FT-IR

The infrared spectra of absorption or emission of the AgNPs and GAE in solution were identified using a Perkin Elmer Spectrum Two FT-IR spectrophotometer (Waltham, MA, USA) in the wavelength range 4000–500 cm^−1^. The baseline corrections were performed for all spectra.

#### 3.3.4. HRTEM

HRTEM analysis was performed by the addition of a drop of each AgNP solution on carbon-coated copper grids, then left to dry under ambient conditions. The shape and size of AgNPs were analyzed using TecnaiF20 HRTEM (FEI Company, Hillsboro, OR, USA) with an accelerating voltage of 300 kV at the Electron Microscope Unit (University of Cape Town, South Africa). In addition, the core size distribution of the AgNPs was calculated using ImageJ software (National Institutes of Health, Bethesda, MD, USA).

### 3.4. Assessment of the Stability of AgNPs

The stability of AgNPs over time was evaluated following a previous method [80], by measuring the UV–Vis profile hourly for 0–6 hr in water, Dulbecco’s phosphate-buffered saline (DPBS; Lonza, Walkersville, MD, USA), and MHB (Sigma). The AgNPs were washed as before, and the pellets were resuspended in the test solutions and then incubated at 37 °C. Their UV–Vis profile (300–650 nm) was measured using a POLARstar Omega plate reader.

### 3.5. Anti-Bacterial Activity of the AgNPs

The anti-bacterial activity of the AgNPs was evaluated using Gram-negative and Gram-positive bacterial strains; i.e., *Klebsiella pneumoniae* (*K. pneumoniae*), *Escherichia coli* (*E. coli*), *Pseudomonas aeruginosa* (*P. aeruginosa*), *Staphylococcus aureus* (*S. aureus*), *Methicillin-resistant Staphylococcus aureus* (*MRSA*), *Staphylococcus epidermidis* (*S. epidermidis*), and *Streptococcus pyogenes* (*S. pyogenes*). All the bacterial strains were purchased from American Type Culture Collection (ATCC, Manassas, USA). The anti-bacterial activity was determined by agar well diffusion and microdilution assays according to the standards set by the Clinical and Laboratory Standard Institute with few modifications [81].

Bacterial colonies were cultured in MHB while shaking at 120 rpm overnight at 37 °C, then diluted at 1:100 in fresh MHB and cultured until reaching a 0.5 McFarland turbidity standard prior to experiments. The bacteria were used for anti-bacterial tests at 1.5 × 10^6^ CFU/mL by diluting the 0.5 McFarland turbid suspensions to 1:150.

#### 3.5.1. Agar Diffusion Assay

The bacterial cultures were streaked on Mueller–Hinton Agar (MHA; Sigma) plates at 1.5 × 10^6^ CFU/mL using sterile cotton swabs. Wells of 6 mm diameter were made on the MHA plates, to which 20 μL of the AgNPs were added. The MHA plates were then incubated overnight at 37 °C. Ciprofloxacin (10 µg/mL; Sigma) was used as a positive control. The anti-bacterial activity of the AgNPs was determined by the presence of clear zones surrounding the wells. The diameter of the clear zones was measured using calipers after 24 hr.

#### 3.5.2. Microdilution Assay

Microdilution assay was used to determine the minimum inhibitory concentration (MIC) [47,49] and minimum bactericidal concentration (MBC) [82] of the AgNPs following previously described protocols.

##### Minimum Inhibitory Concentration (MIC)

The MIC of the AgNPs required to inhibit the visual growth of the bacteria was determined according to the microdilution method [47,49]. The bacteria (1.5 × 10^6^ CFU/mL) were exposed to different concentrations (0–100 μg/mL) of the AgNPs and incubated at 37 °C for 24 hr. The MIC values were visually observed, followed by measuring the optical density (OD) of the bacterial culture at 600 nm using a POLARstar plate reader. Bacterial growth was further evaluated by Alamar Blue colorimetric assay (Invitrogen, Eugene, Oregon, USA), where 10 μL of the dye was added to each well and incubated for 3 hr. The blue color was converted to a pink-purplish color by live bacteria that was quantifiable by measuring absorbance at 570 nm and a reference wavelength at 700 nm [83].

##### Minimum Bactericidal Concentration (MBC)

The MBC of the AgNPs was determined in the bacteria used for MIC, where a loopful of broth from the wells was spotted onto fresh MHA and incubated at 37 °C for 24 hr. The lowest concentration that exhibited no growth on the MHA was considered as the MBC.

### 3.6. Cytotoxicity Assay of the AgNPs

The effect of the AgNPs was evaluated by MTT assay, as previous described, on the human cell lines, KMST-6 normal skin fibroblasts, HT-29 and CaCo-2 colon carcinoma cells [80]. The cells were purchased from ATCC, and were cultured in Dulbecco’s Modified Eagle’s Medium (DMEM, Lonza) supplemented with 10% fetal bovine serum (Gibco, Waltham, Massachusetts, USA) and 1% penicillin-streptomycin cocktail (Lonza) and incubated at 37 °C. The cells were then seeded in 96 well plates at 1 × 10^5^ cell/mL density, 100 µL in each well and incubated for 24 hr. The cells were treated with 0–500 μg/mL of the AgNPs and extracted in triplicates. The cell viability was assessed by adding 10 μL of 5 mg/mL of MTT (Sigma) solution to each well and incubated for 3 hr. Later, the MTT solution was discarded and 100 μL of DMSO was added to each well. The absorbance of the formazan product was measured at 570 nm with a reference at 700 nm using a POLARstar Omega plate reader. The concentration that inhibited 50% cell growth (IC_50_) was further analyzed by Graphpad Prism 6.0.

### 3.7. Statistical Analysis

All the experiments were carried out in triplicate and the results were analysed using Graphpad Prism 6.0. The data are presented as means ± SD according to one-way ANOVA test followed by a post hoc, multiple comparisons (Tukey’s) test. A *p*-value of <0.05 was considered statistically significant.

## 4. Conclusions

The growing interest in medical application of AgNPs has warranted greener methods for their synthesis to prevent toxicity and improve biocompatibility. Synthesis of AgNPs using plant extracts presents not only a greener method but a sustainable, reproducible and upscalable approach. However, for biomedical applications, the safety of plant-synthesized AgNPs must be authenticated. The current study demonstrated that GAE alone, and in the presence of a chemical reducing agent, produced AgNPs with distinct bioactivities. The GA-AgNPs demonstrated broad spectrum anti-bacterial effects on both Gram-positive and Gram-negative bacteria, and non-selective cytotoxicity on normal and colon cancer cells in the same concentration range. Interestingly, these effects were reduced in the GAC-AgNPs, suggesting that surface coating can be used to channel the effects of AgNPs. The selective and reduced cytotoxicity demonstrated by GAC-AgNPs towards colon cancer cells demonstrated that surface composition can be used to control the biodistribution, uptake and efficacy of AgNPs. AgNPs represent the next generation of antimicrobial agents, and have potential to help solve the antimicrobial resistance problem. Their biocompatibility can be enhanced by modifying the surface of AgNPs with targeting molecules or biocompatible molecules, such as PEG, for medical application.

## Figures and Tables

**Table 1:** Phytochemical analysis and antioxidant capacity of GAE.

Phytochemical Content	4 mg/mL GAE
Flavanols (mg/g)	0.0187
Flavonols (mg/g)	0.0019
TPC (mgGAE/g)	0.0003
DPPH (µmolTE/g)	0.0000
ORAC (µmolTE/g)	0.0000
FRAP (µmolAAE/g)	0.0000

**Table 2:** Physicochemical properties of the AgNPs.

AgNPs	λmax/SPR (nm)	Core Size (nm)	Hydrodynamic Size (nm)	ζ-Potential (mV)	Pdi
C-AgNPs	408	10 ± 1.69	87.22 ± 5.94	−30.50 ± 4.63	0.30 ± 0.03
GAC-AgNPs	414	12 ± 0.61	144.39 ± 4.99	+9.33 ± 17.23	0.55 ± 0.01
GA-AgNPs_0.4g	416	12 ± 0.47	76.21 ± 6.35	−29.60 ± 1.90	0.28 ± 0.03
GA-AgNPs_0.5g	414	12 ± 0.25	94.62 ± 10.06	−27.07 ± 3.71	0.23 ± 0.06

**Table 3:** Anti-bacterial activity of the synthesized AgNPs.

Treatments	ZOI (mm)				
	*S. aureus*	*MRSA*	*S. epidermidis*	*K. pneumoniae*	*E. coli*
MHB	0	0	0	0	0
GAE	0	0	0	0	0
C-AgNPs	9.8	9.8	8.4	11	6.2
GAC-AgNPs	0	0	0	0	0
GA-AgNPs_0.4g	14.2	13.8	20	13.6	11.2
GA-AgNPs_0.5g	13	9.8	19	14.6	10.2

**Table 4:** MIC of the AgNPs on test bacteria.

Treatments	MIC (µg/mL)					
	*S. aureus*	*MRSA*	*S. epidermidis*	*S. pyogenes*	*K. pneumoniae*	*E. coli*
GAE	>100	>100	>100	>100	>100	>100
C-AgNPs	>100	>100	>100	>100	>100	>100
GAC-AgNPs	>100	>100	>100	>100	>100	>100
GA-AgNPs_0.4g	6.25	6.25	6.25	6.25	6.25	25
GA-AgNPs_0.5g	6.25	6.25	6.25	6.25	6.25	6.25

**Table 5:** MBC of the AgNPs on test bacteria.

Treatments	MBC (µg/mL)					
	*S. aureus*	*MRSA*	*S. epidermidis*	*S. pyogenes*	*K. pneumoniae*	*E. coli*
GAE	>100	>100	>100	>100	>100	>100
C-AgNPs	>100	>100	>100	>100	>100	>100
GAC-AgNPs	>100	>100	>100	>100	>100	>100
GA-AgNPs_0.4g	>100	12.5	100	>100	25	12.5
GA-AgNPs_0.5g	100	12.5	25	>100	12.5	12.5

**Table 6:** The IC_50_ values of treatments against different cell lines.

Cell-Lines	IC_50_ (µg/mL)				
	GAE	C-AgNPs	GAC-AgNPs	GA-AgNPs_0.4g	GA-AgNPs_0.5g
KMST-6	>100	87.40	>100	0.67	0.90
Caco-2	>100	41.67	92.00	0.82	1.26
HT-29	>100	50.54	>100	1.16	1.55

## Data Availability

The data presented in the study can be requested from the authors.
